# Nutritional Behaviors of Patients With Heart Failure

**DOI:** 10.1155/nrp/1519211

**Published:** 2025-03-29

**Authors:** Canan Demir Barutcu, Serap Gokce Eskin

**Affiliations:** ^1^Faculty of Health Science, Medical Nursing Department, Mehmet Akif Ersoy University, Burdur, Turkey; ^2^Faculty of Nursing, Aydin Adnan Menderes University, Aydin, Turkey

**Keywords:** anthropometric measurements, dietary behaviors, heart failure, nutritional status

## Abstract

The study aimed to evaluate the nutritional behavior of patients with heart failure in accordance with nutrition guidelines. This was an analytical cross-sectional study and conducted at the cardiology unit of a university hospital. The study sample consisted of 104 patients. We concluded that middle-aged patients, who had Stage I heart failure, whose fat percentage was 40%–59%, and whose metabolic age was young were found to be nourished by nutrition guidelines. It is recommended that the nutritional behaviors of heart failure patients be monitored and nursing interventions planned according to the guidelines.

**Summary**

• Patients with heart failure are often not fed according to guideline recommendations.
• NYHA Stage IV patients do not eat following the guide recommendations.
• It should be known whether heart failure patients are fed according to the guidelines, and their nutritional status and behavior should be monitored.
• Based on the findings of this study, awareness can be raised that patients with heart failure should be evaluated by nurses at every visit to ensure that they have correct eating habits.

## 1. Introduction

Heart failure (HF) is a disease with an increasing incidence and prevalence that impairs quality of life and has a poor prognosis despite developments in medicine [1, 2]. The American Heart Association (AHA) predicts that more than eight million individuals above 18 years will have HF by 2030 [1].

Self-care is critical for HF management, and self-care management programs are recommended to patients with HF. In self-care maintenance, patients are kept in a compensatory state by symptom monitoring, diet intake according to recommendations, and medication adherence [3]. Patients with HF are also in the risk group for malnutrition which is an independent predictor for HF and mortality [4, 5]. The negative nitrogen balance, increased catabolism, and protein loss that develops throughout HF lead to malnutrition. Venous congestion resulting from HF leads to conditions that hinder nutrition such as loss of appetite, nausea, vomiting, and early satiety [2, 6, 7]. The cardiac cachexia that develops because of all these factors significantly reduce the quality of life and chance of survival [6–9]. Studies indicate that about one-third or more of HF patients have malnutrition [6, 10, 11]. The maintenance of sufficient nutrition is particularly important for improving health outcomes [12]. Patients should be regularly evaluated for the prevention and correction of malnutrition [2, 7]. The quality of life of the patients may be improved through a proper nutrition program in collaboration with the medical team. In the present study, the aim was to evaluate patients treated for HF concerning their nutritional behavior by nutrition guidelines through a nutrition scale.

## 2. Methods

This was an analytical cross-sectional study conducted with 104 eligible patients at the Cardiology Clinic of An University Hospital between November 2021 and April 2022. The number of CHF patients followed for six months was 126; all patients (*n* = 104) who met the research criteria were included in the study. The criteria included in the sample included being diagnosed with HF at least 6 months ago, being a middle-aged (min:45) or elderly HF patient, being able to communicate, and participating on a volunteer basis. Data for patient characteristics form and the Scale for Dietary Behaviors in Heart Failure (SDBHF) were collected with face-to-face interviews. Data collection took approximately 30 min.

A patient information form developed by the researchers was used. The form consisted of a total of seven questions regarding age, gender, marital status, educational status, smoking status, type of job, and the patient's stage of HF. The SDBHF was developed by Boy and Enç [7] with the aim of evaluating dietary behaviors in HF patients; it included four subdimensions and 19 items of the 4-Likert type [7]. The scale contained the health habits, the salt restriction, the sugar restriction, and the prevention of retention subdimension. A score of ≤ 46 indicated that the patient was not nourished in accordance with guideline recommendations. Increasing scores indicated better compliance with the guidelines. Scores of 19 ≤ and < 46 indicated that the patient was not nourished in accordance with the recommendations of the guidelines and expert academicians, scores between 46 ≤ and < 51 indicated that the patient was nourished to some extent, scores between 51 ≤ and < 55 indicated that the patient was nourished moderately;, and scores between 55 ≤ and ≤ 76 indicated that the patient was nourished strongly considered the guidelines and recommendations of expert academicians. Cronbach's alpha confidence coefficient was found to be 0.72. In the present study, Cronbach's alpha coefficient was found to be 0.78. Anthropometric measurements were done by the researcher. Body weight was expressed as “kg” and height as “cm.” Measurements were done by using a portable bioelectrical impedance analysis (BIA) device from the Nutrition and Dietetics Department in the XXX University Health Sciences Faculty. BIA was performed by using the foot-to-foot method with an impedance analyzer (Body Composition Analyzer, Tanita Inc. Tokyo, Japan, Model TBF 300). BMI, body fat percentage, body fat mass, and lean body mass were automatically output from the impedance analyzer. Height was measured with a portable 140–200-cm capacity, and Tartı brand stadiometer provides height measurement in 1 mm intervals. Anthropometric measurements were made in the morning and while the patient was fasting. The data of the study were analyzed using the IBM Statistical Package for the Social Sciences (SPSS) 22 program. The number and percentage distributions of the descriptive data of the patients were stated. For parametric variables between the descriptive characteristics of the patients and the SDBHF and anthropometric measurements, an independent sample *t* test was performed, and in nonparametric variables, the Kruskal–Wallis, Mann–Whitney-U, and one-way ANOVA tests were performed. The Pearson correlation test was used to determine the relationship between the mean scores of the SDBHF and the anthropometric measurements. In the correlation analysis, *r* = 0.00–0.24 was evaluated as weak, *r* = 0.25–0.49 moderate, *r* = 0.50–0.74 strong, and *r* = 0.75–1.00 very strong. The significance level was accepted as *p* < 0.05.

Approval was obtained from the researchers who developed the scale. The approval to conduct the study was obtained from the XXX University Faculty of Health Science Noninterventional Research Ethics Committee (approval no. 2019/092, 25.12.2019). Institutional permission was obtained from the hospital for the study. After the patients had been informed about the purpose of the study, written and verbal consent was obtained from the volunteer participants. The study was conducted in accordance with the principles of the Helsinki Declaration.

## 3. Results

The mean age of the patients was 70.43 + 9.19 years, 66.4% were female, 55.4% were between the ages of 60–74, 69.3% were married, 81.2% were primary school graduates, and 52% were housewives. The mean score of the SDBHF was found to be 47.18 + 6.21 (Table 1).

A correlation analysis was performed between the SDBHF and the mean scores of the subdimensions. A strong positive correlation was detected between the total scale score and the healthy habits subdimension, and a moderate positive correlation was found between salt restriction and sugar restriction subdimension mean scores. There was a positive weak relationship between the healthy habits subdimension and the salt restriction subdimension; a positive moderate relationship was found between sugar restriction and SDBHF. A weak positive correlation was found between the subdimension of preventing retention and the salt restriction subdimension (Table 2).

A statistically significant difference was found in the nutritional status in accordance with the guidelines according to age, and this difference was found to have resulted from the 45–59 age group. Of the participants, 75.2% had Stage 3 HF. The nutritional status in accordance with the nutritional guidelines was found to be statistically significantly higher in the patients with Stage 1 HF (Table 3).

The body mass index of 52.5% of the patients was found to be between 25 and 29.99. The fat percentage of 55.7% of the patients was between 25% and 39%, the water percentage was found to be between 45 and 59 in 66.3% of the patients, and the muscle percentage was found to be between 45 and 59 in 51.5% of the patients. The metabolic age of 57.4% of the patients was found to be 55–69 years old. When the dietary behaviors were evaluated, the dietary behaviors of the patients with a muscle percentage of 60% and above were higher than the others, and the difference was statistically significant. Patients with a fat percentage of 40%–54% and patients with a metabolic age below 50 also had a higher mean nutritional behavior score, and the difference was statistically significant (Table 3).

## 4. Discussion

There is a strong relationship between nutrition and HF [4]. In this study, the nutritional behavior of HF patients was evaluated by comparing this behavior with some anthropometric measurements.

According to the mean scores of SDBHF, participants were seen to moderately comply with nutrition guidelines and the recommendations of experts. Pressler et al. [11] reported that one-third of women with HF do not comply with a therapeutic diet [11]. Studies report that nutrition problems are common in patients with HF [12–16].

In our study, participants below 60 were detected to be nourished in accordance with the guidelines and recommendations of experts. In a study, the malnutrition risk was found to be lower in younger patients [17]. HF is a disease with social, psychological, and behavioral aspects in addition to physical functions [18]. Most of the patients with HF are older, and advanced age is related to the consumption of a low-quality diet [5]. Nutritional problems may develop with aging due to reduced muscle power and muscle mass, physical weakness, functional impairment, and a lack of social support.

In our study, Stage 1 patients, according to the functional classification of New York Heart Association (NYHA), were found to be nourished by considering the guidelines and recommendation of experts. It is known that NYHA Stage 1 patients do not have any physical activity restrictions. These patients can eat in accordance with the recommendations as they do not have any problems in preparation and consumption [15].

According to the results of the study, patients with a fat percentage of 10%–24% partly complied with the recommendations of guidelines and experts. The fat percentage should be between 14% and 24% in a healthy individual. This was an expected result.

In our study, patients whose muscle mass was above 60% were found to be fed in accordance with the nutrition guidelines. For patients with HF, diets that contain sufficient protein, fiber, and restricted salt and fluid are recommended [14, 19, 20]. Patients who are fed in accordance with these recommendations are expected to have a normal fat and muscle mass [20–22]. Our finding supports this information.

When the subdimensions were analyzed, there was a strong positive correlation between all subdimensions and the total scale score, except for the prevention of retention subdimension. Restricted consumption of salt and sugar, healthy eating habits, and exercise are the disease management strategies recommended by experts in patients with HF [23–25]. It is expected that these factors are related to each other in individuals who are fed in accordance with with the recommendations of experts and guidelines.

## 5. Conclusions

In our study, it was found that patients with HF who are in the middle-age group, who have Stage 1 HF, a fat percentage of 40%–59%, and a low metabolic age were fed in accordance with the nutritional guidelines. Nutrition is an important parameter for survival in patients with HF. The purpose of nutrition in HF is to prevent water retention and edema and to provide adequate and balanced nutrition. Patients may be obese in the first stage of HF, and cachexia may occur in advanced stages. It is known that cachexia increases mortality, and it is also known that obesity protects HF patients from mortality but is also a risk factor. It is important that nurses, who are primarily responsible for the care of the patient, consider the patient with a holistic perspective. It should be known whether HF patients are fed according to the guidelines, and their nutritional status and behavior should be monitored.

## Figures and Tables

**Table 1:** Comparison of the scale for dietary behaviors in heart failure mean scores according to individual characteristics.

Individual characteristics	*n*	%	*X* ± SD	Statistical analysis
Gender				
Female	68	66.4	47.44 ± 5.90	*t* = 0.556
Male	36	33.6	46.69 ± 6.81	*p*=0.580
Age				
45–59	13	11.9	50.84 ± 4.57	*F* = 2.781
60–74	59	55.4	46.44 ± 5.94	*p*=0.047^∗^
75–90	32	32.7	47.06 ± 6.87	
Marital status				
Married	70	67.3	41.20 ± 6.07	*t* = 0.408
Single	34	32.7	38.96 ± 5.09	*p*=0.685
Educational status				
Primary school	83	81.2	47.21 ± 5.79	*F* = 0.328
High school	10	8.9	45.90 ± 9.37	*p*=0.721
University	11	9.9	48.09 ± 6.21	
Smoking				
Yes	39	37.5	47.15 ± 6.67	*t* = −0.036
No	65	62.5	47.20 ± 5.97	*p*=0.972
Working status				
Retired	51	48.5	47.45 ± 6.60	*t* = 0.429
Housewife	53	51.5	46.92 ± 5.86	*p*=0.669
NYHA				
I	15	13.9	52.26 ± 5.36	*F* = 4.710
II	7	5.0	48.57 ± 6.42	*p*=0.004^∗^
III	76	75.2	46.15 ± 6.01	
IV	6	5.9	45.83 ± 4.83	

*Note: X* = mean; *t* = Student's *t*-tests; *F* = one-way ANOVA.

Abbreviation: SD = standard deviation.

⁣^∗^*p* < 0.05.

**Table 2:** Examination of the relationship between the scale for dietary behaviors in heart failure and subdimensional scores.

**Scale and subdimensions**		**SDBHF**	**Healthy habits**	**Salt restriction**	**Sugar restriction**	**Prevention of fluid retention**

Scale for Dietary Behaviors (SDBHF)	*r*	1	0.767	0.645	0.623	0.161
	*p*		0.000	0.000	0.000	0.102

Healthy habits	*r*		1	0.234	0.302	−0.038
	*p*			0.017	0.002	0.705

Salt restriction	*r*			1	0.375	0.284
	*p*				0.000	0.004

Sugar restriction	*r*				1	−0.077
	*p*					0.434

Prevention of fluid retention	*r*					1
	*p*					

*Note: r* = Pearson's correlation.

⁣^∗^*p* < 0.05.

**Table 3:** Comparison of patients' anthropometric measurements according to the mean scores of the Scale for Dietary Behaviors in Heart Failure.

Anthropometric measurements	*n*	%	*X* ± SD	Statistical analysis
BMI (kg/m^2^)				
18–24.99	31	30.7	47.18 ± 6.72	*F* = 0.001
25–29.99	55	52.5	47.16 ± 6.33	*p*=0.999
> 30	17	16.8	47.23 ± 5.04	
Fat (%)				
10–24	19	18.4	49.78 ± 6.94	*F* = 3.067
25–39	58	55.7	45.98 ± 5.94	*p*=0.050^∗^
40–54	27	25.9	47.92 ± 5.75	
Water (%)				
30–44	10	9.9	44.10 ± 5.27	*F* = 1.915
45–59	70	66.3	47.12 ± 6.12	*p*=0.153
> 60	24	23.8	48.62 ± 6.56	
Muscle (%)				
30–44	36	35.6	46.63 ± 5.77	*F* = 3.724
45–59	54	51.5	46.48 ± 6.29	*p*=0.028^∗^
> 60	14	12.9	51.28 ± 5.78	
Metabolic age (year)				
40–54	18	15.9	52.16 ± 4.93	*F* = 8.213
55–69	58	57.4	46.13 ± 5.63	*p*=0.000^∗^
70–84	28	26.7	45.53 ± 6.65	

*Note: X* = mean; *F* = one-way ANOVA.

Abbreviations: BMI = body mass index, SD = standard deviation.

⁣^∗^*p* < 0.05.

## Data Availability

No data are available.
